# Supplementary material for: Sex differences in the longitudinal relationship of low-grade inflammation and echocardiographic measures in the Hoorn and FLEMENGHO Study
Source: PLoS One. 2021 May 4;16(5):e0251148. doi: 10.1371/journal.pone.0251148 (PMC8096104; doi:10.1371/journal.pone.0251148)
Supplement: S1 Table — (PDF) [file pone.0251148.s001.pdf]

S1 Table. Baseline characteristics of complete cases (N=383/491) versus loss to follow-up (N=346/156) of the Hoorn and FLEMENGHO Study participants.

	Hoorn Study			FLEMENGHO		
	Complete cases (N=383)	Loss to follow-up (N=346)	P-value	Complete cases (N=491)	Loss to follow-up (N=156)	P-value
Age, years	66.4±6.1	70.8±6.1	0.20	49.1±14.8	48.8±18.2	0.85
Female	181 (47.3%)	181 (52.3%)	< 0.001	248 (50.5%)	80 (51.3%)	0.94
BMI, kg/m^2	27.3±3.5	27.6±4.1	0.29	26.5±4.2	26.3±4.6	0.85
Glucose metabolism status			< 0.001			1.00
Normal glucose metabolism	178 (46.5%)	108 (31.2%)		N/A	N/A	
Impaired glucose metabolism	91 (23.8%)	108 (25.1%)		N/A	N/A	
Type 2 Diabetes	112 (29.2%)	148 (42.8%)		17 (3.5%)	6 (3.8%)	
Systolic blood pressure, mmHg	139±19	145±21	< 0.001	127±16	129±18	0.34
Diastolic blood pressure, mmHg	83±11	83±11	0.96	80±9	79±10	0.32
Hypertension	197 (51.4%)	215 (62.1%)	0.005	120 (24.4%)	41 (26.3%)	0.72
Current smoker	58 (15.1%)	67 (19.4%)	0.27	89 (18.1%)	37 (23.7%)	0.31

eGFR, mL/min/1.73m^2	82.8±12.4	79.9±14.8	0.004	103±25	107±25	0.32
History of cardiovascular disease	182 (47.5%)	204 (59.0%)	<0.001	20 (4.1%)	17 (10.9%)	0.003
Low-grade inflammation						
CRP, mg/L	2.0 [1.0;3.9]	2.6 [1.4;5.4]	< 0.001	1.2 [0.9;2.2]	1.3 [0.9;2.6]	0.65
Serum amyloid A, mg/L	1.6 [1.0;2.9]	1.9 [1.2;3.7]	0.002	N/A	N/A	N/A
IL-6, ng/L	1.4 [1.0;2.1]	1.6 [1.1;2.4]	< 0.001	1.4 [1.0;2.0]	1.5 [1.0;2.8]	0.02
IL-8, ng/L	13.8 [10.8;18.2]	15.0 [11.5;19.0]	0.07	6.9 [5.3;9.6]	7.1 [5.3;10.2]	0.59
sICAM-1, µg/L	249±56	273±69	< 0.001	235±81	275±104	< 0.001
TNF-α, ng/L	8.1 [6.8;9.7]	8.4 [7.0;10.0]	0.26	2.1 [1.8;2.6]	2.1 [1.7;2.7]	0.63
Endothelial dysfunction						
sICAM-1, µg/L	249±56	273±69	< 0.001	235±81	275±104	< 0.001
sVCAM-1, µg/L	381 [337;436]	416 [362;477]	< 0.001	501±177	583±254	< 0.001
sE-selectin, µg/L	19.2±8.1	19.6±8.3	0.48	14.8 [10.8;19.4]	16.5 [12.4;21.4]	0.01
sTM, µg/L	3.5±0.8	3.5±1.0	0.14	N/A	N/A	N/A

Echocardiographic measures						
LVEF, %	62.1±7.9	60.9±8.7	0.06	68.6±7.0	67.5±8.1	0.13
LVMI, g/$m^{2.7}$	40.4±11.6	44.9±15.4	< 0.001	41.3±10.4	40.9±12.5	0.68
LAVI, mL/m^2	24.6±8.0	27.5±11.9	< 0.001	23.1±6.5	21.8±6.7	0.04

Values are depicted as numbers (percentages); means±standard deviations; medians [interquartile ranges].

Analysis methods: Pearson Chi-Square, independent samples T-test and Mann-Whitney U test.

Abbreviations: FLEMENGHO = Flemish Study on Environment, Genes and Health Outcomes, BMI = body mass index, GMS = glucose metabolism status, NGM = normal glucose metabolism, IGM = impaired glucose metabolism, T2D = type 2 diabetes, SBP = systolic blood pressure, DBP = diastolic blood pressure, eGFR = estimated glomerular filtration rate, CVD = cardiovascular diseases, CRP = C-reactive protein, SAA = serum amyloid A, IL-6 = interleukin-6, IL-8 = interleukin-8, sICAM1 = soluble intercellular adhesion molecule 1, TNFa = tumor necrosis factor α, sVCAM1 = soluble vascular adhesion molecule 1, sE-selectin = soluble endothelial selectin, sTM = soluble thrombomodulin.
