# Supplementary material for: Sex differences in the longitudinal relationship of low-grade inflammation and echocardiographic measures in the Hoorn and FLEMENGHO Study
Source: PLoS One. 2021 May 4;16(5):e0251148. doi: 10.1371/journal.pone.0251148 (PMC8096104; doi:10.1371/journal.pone.0251148)
Supplement: S2 Table — (PDF) [file pone.0251148.s002.pdf]

S2 Table. 'Longitudinal' and between person associations of separate biomarkers on cardiac structure and function measures in the Hoorn Study and FLEMENGHO.

	Hoorn						FLEMENGHO					
	Total (N=289)		Female (N=137)		Male (N=152)		Total (N=315)		Female (N=159)		Male (N=156)	
LVEF, %	Within person	Between persons	Within person	Between persons	Within person	Between persons	Within person	Between persons	Within person	Between persons	Within person	Between persons
Low-grade inflammation												
CRP	-0.7 (-2.1;0.6)	0.02 (-1.0;1.1)	-0.1 (-2.2;2.0)	0.4 (-1.1;1.8)	-0.7 (-2.4;1.1)	-0.2 (-1.6;1.1)	0.6 (-0.1;1.3)	**0.9 (0.1;1.7)**	0.9 (-0.1;1.9)	0.8 (-0.3;1.9)	0.3 (-0.7;1.3)	1.0 (-0.3;2.2)
SAA	-0.5 (-1.8;0.7)	-0.1 (-1.0;0.9)	0.5 (-1.9;2.8)	-0.1 (-1.6;1.5)	-0.6 (-2.0;0.8)	0.2 (-1.1;1.4)	N/A	N/A	N/A	N/A	N/A	N/A
IL-6	0.4 (-0.8;1.6)	0.1 (-0.9;1.1)	0.9 (-1.0;2.7)	0.3 (-1.1;1.7)	0.8 (-0.9;2.4)	-0.1 (-1.6;1.3)	0.5 (-0.2;1.2)	0.4 (-0.4;1.2)	0.5 (-0.6;1.5)	1.0 (-0.1;2.1)	0.5 (-0.5;1.5)	-0.3 (-1.5;0.8)
IL-8	0.4 (-1.0;1.9)	-0.5 (-1.4;0.4)	-0.7 (-2.9;1.5)	-1.0 (-2.1;0.2)	1.7 (-0.2;3.6)	0.6 (-0.9;2.0)	-0.4 (-1.2;0.3)	-0.3 (-1.1;0.6)	-0.8 (-1.9;0.4)	-0.6 (-1.7;0.5)	-0.2 (-1.2;0.7)	0.2 (-1.0;1.4)
sICAM-1	-0.6	0.1	**-2.6**	0.3	0.5	-0.2	-0.8	-0.3	0.4	-0.5	**-1.2**	-0.2

	(-2.1;0.9)	(-1.0;1.1)	**(-5.1;-0.02)**	(-1.2;1.9)	(-1.3;2.2)	(-1.5;1.3)	(-1.6;0.1)*	(-1.2;0.7)*	(-1.4;2.1)	(-1.9;0.9)	**(-2.2;-0.2)**	(-1.4;1.1)
TNF-α	-0.2	-0.5	**-2.2**	-0.6	**2.2**	-0.6	-0.2	-0.7	-0.2	**-1.1**	-0.2	0.1
	(-1.7;1.4)*	(-1.4;0.5)*	**(-4.5;0.01)**	(-1.9;0.7)	**(-0.01;4.3)**	(-1.9;0.8)	(-1.0;0.5)	(-1.5;0.03)	(-1.2;0.8)	**(-2.1;-0.1)**	(-1.3;0.9)	(-1.1;1.2)
Endothelial dysfunction												
sICAM-1	-0.6	0.1	**-2.6**	0.3	0.5	-0.2	-0.8	-0.3	0.4	-0.5	**-1.2**	-0.2
	(-2.1;0.9)	(-1.0;1.1)	**(-5.1;-0.02)**	(-1.2;1.9)	(-1.3;2.2)	(-1.5;1.3)	(-1.6;0.1)*	(-1.2;0.7)*	(-1.4;2.1)	(-1.9;0.9)	**(-2.2;-0.2)**	(-1.4;1.1)
vCAM-1	-0.8	0.3	-1.9	0.8	0.5	-0.2	0.03	-0.4	0.1	-0.5	-0.1	-0.2
	(-2.4;0.8)	(-0.6;1.2)	(-4.3;0.4)	(-0.6;2.3)	(-1.7;2.7)	(-1.4;1.0)	(-0.7;0.7)	(-1.1;0.3)	(-1.0;1.2)	(-1.5;0.4)	(-1.0;0.8)	(-1.2;0.7)
E-selectin	1.0	0.8	0.3	0.3	1.5	1.0	**-1.1**	-0.2	0.1	-0.3	**-1.6**	-0.1
	(-0.7;2.8)	(-0.2;1.8)	(-2.6;3.2)	(-1.3;2.0)	(-0.7;3.7)	(-0.3;2.3)	**(-2.1;-0.1)***	(-0.9;0.60*	(-1.9;2.0)	(-1.4;0.9)	**(-2.7;-0.5)**	(-1.2;1.0)
sTM	-0.4	0.7	-1.5	1.2	0.2	0.3	N/A	N/A	N/A	N/A	N/A	N/A
	(-1.8;1.0)	(-0.2;1.6)	(-3.8;0.7)	(-0.2;2.6)	(-1.6;2.0)	(-0.9;1.4)						
LVMI, $g/m^{2.7}$	Total (N=304)		Female (N=146)		Male (N=158)		Total (N=477)		Female (N=242)		Male (N=235)	
	Within person	Between persons	Within person	Between persons	Within person	Between persons	Within person	Between persons	Within person	Between persons	Within person	Between persons

Low-grade inflammation												
CRP	-0.2 (-1.6;1.2)	-0.5 (-1.6;0.7)	-0.1 (-2.0;1.9)	-0.3 (-1.8;1.4)	-0.5 (-2.5;1.5)	-0.7 (-2.3;1.0)	0.04 (-0.5;0.6)	0.4 (-0.5;1.2)	-0.2 (-0.9;0.5)	-0.3 (-0.5;1.6)	0.4 (-0.4;1.1)	0.5 (-0.9;1.9)
SAA	-0.4 (-1.7;0.9)	-0.6 (-1.7;0.5)	-1.2 (-3.2;0.8)	-0.7 (-2.4;1.0)	-0.03 (-1.7;1.6)	-0.6 (-2.1;0.9)	N/A	N/A	N/A	N/A	N/A	N/A
IL-6	0.7 (-0.5;1.9)	-0.2 (-1.4;1.0)	0.6 (-1.0;2.2)	-0.7 (-2.3;0.8)	0.5 (-1.3;2.3)	0.3 (-1.5;2.1)	-0.1 (-0.6;0.5)	-0.3 (-1.1;0.5)	0.2 (-0.5;0.9)	0.2 (-0.8;1.3)	-0.3 (-1.1;0.5)	-0.6 (-1.7;0.6)
IL-8	-0.3 (-1.7;1.2)	-0.3 (-1.3;0.8)	0.4 (-1.6;2.4)	0.2 (-1.0;1.3)	-0.5 (-2.6;1.5)	-1.2 (-3.1;0.6)	0.4 (-0.2;0.9)	0.2 (-0.6;0.9)	0.2 (-0.6;0.9)	-0.03 (-1.0;1.0)	0.5 (-0.2;1.3)	0.4 (-0.8;1.6)
sICAM-1	-0.1 (-1.6;1.5)	-0.1 (-1.3;1.0)	-1.2 (-3.5;1.2)	1.0 (-0.6;2.6)	0.8 (-1.3;2.9)	-1.1 (-2.7;0.6)	-0.1 (-0.6;0.5)	-0.7 (-1.6;0.2)	0.3 (-0.9;1.5)	-0.2 (-1.5;1.2)	-0.1 (-0.8;0.5)	-1.1 (-2.4;0.1)
TNF-α	0.6 (-1.1;2.3)	0.2 (-0.9;1.3)	**1.7 (-0.6;3.9)**	**1.6 (0.1;3.1)**	0.5 (-2.2;3.2)	-1.4 (-3.1;0.4)	0.5 (-0.1;1.0)	-0.3 (-1.1;0.4)	0.5 (-0.3;1.2)	-0.9 (-1.9;0.1)	0.4 (-0.4;1.2)	0.2 (-0.9;1.4)
Endothelial dysfunction												

sICAM-1	-0.1 (-1.6;1.5)	-0.1 (-1.3;1.0)	-1.2 (-3.5;1.2)	1.0 (-0.6;2.6)	0.8 (-1.3;2.9)	-1.1 (-2.7;0.6)	-0.1 (-0.6;0.5)	-0.7 (-1.6;0.2)	0.3 (-0.9;1.5)	-0.2 (-1.5;1.2)	-0.1 (-0.8;0.5)	-1.1 (-2.4;0.1)
vCAM-1	-1.1 (-2.7;0.6)	-0.1 (-1.2;1.0)	-0.6 (-2.9;1.6)	1.0 (-0.6;2.6)	-1.1 (-3.5;1.4)	-0.9 (-2.4;0.6)	0.1 (-0.4;0.6)	-0.4 (-1.0;0.3)	0.1 (-0.6;0.7)	-0.2 (-1.1;0.7)	0.2 (-0.4;0.9)	-0.6 (-1.6;0.4)
E-selectin	0.7 (-1.3;2.8)	-0.2 (-1.4;0.9)	-0.6 (-3.6;2.5)	1.0 (-0.7;2.8)	1.5 (-1.3;4.2)	-1.0 (-2.5;0.5)	0.1 (-0.6;0.8)	-0.7 (-1.5;0.1)	-0.2 (-1.1;1.5)	-0.9 (-2.0;0.1)	0.04 (-0.8;0.8)	-0.6 (-1.8;0.5)
sTM	**1.5 (-0.01;3.0)**	-0.3 (-1.2;0.7)	1.4 (-0.7;3.5)	0.7 (-0.9;2.3)	**2.1 (0.1;4.2)**	-0.8 (-2.0;0.5)	N/A	N/A	N/A	N/A	N/A	N/A
LAVI, mL/m^2	Total (N=318)		Female (N=150)		Male (N=168)		Total (N=484)		Female (N=245)		Male (N=238)	
	Within person	Between persons	Within person	Between persons	Within person	Between persons	Within person	Between persons	Within person	Between persons	Within person	Between persons
Low-grade inflammation												
CRP	-0.4 (-1.6;0.7)	-0.1 (-1.3;1.2)	0.6 (-1.1;2.3)	-0.2 (-1.8;1.5)	-1.4 (-2.8;0.1)	-0.2 (-2.0;1.7)	-0.1 (-0.4;0.3)	0.1 (-0.5;0.7)	-0.1 (-0.5;0.4)	0.3 (-0.4;1.1)	-0.2 (-0.7;0.4)	-0.1 (-1.2;1.0)
SAA	-0.9	0.2	0.3	0.4	**-1.6**	0.2	N/A	N/A	N/A	N/A	N/A	N/A

	(-1.9;0.1)*	(-1.0;1.5)*	(-1.5;2.1)	(-1.5;2.2)	**(-2.7;-0.4)**	(-1.5;1.8)						
IL-6	-0.1	0.3	0.8	-0.5	-1.1	1.1	-0.1	-0.4	-0.1	-0.3	-0.2	-0.4
	(-1.1;1.0)	(-1.0;1.6)	(-0.7;2.2)	(-2.0;1.0)	(-2.6;0.3)	(-0.9;3.1)	(-0.5;0.3)	(-0.9;0.2)	(-0.6;0.4)	(-1.1;0.5)	(-0.8;0.4)	(-1.3;0.5)
IL-8	0.4	0.6	0.6	-0.3	0.1	1.4	0.1	0.01	0.2	-0.5	0.1	0.5
	(-0.9;1.6)	(-0.7;2.0)	(-1.3;2.5)	(-2.1;1.5)	(-1.5;1.8)	(-0.6;3.4)	(-0.3;0.5)	(-0.6;0.6)	(-0.3;0.7)	(-1.2;0.2)	(-0.5;0.6)	(-0.4;1.4)
sICAM-1	0.3	1.2	0.3	-0.7	0.2	**2.4**	0.1	-0.1	0.6	-0.4	-0.02	-0.2
	(-1.1;1.6)	(-0.1;2.5)	(-2.2;2.7)	(-2.5;1.1)	(-1.4;1.8)	**(0.6;4.2)**	(-0.3;0.6)	(-0.8;0.6)	(-0.2;1.4)	(-1.4;0.6)	(-0.5;0.5)	(-1.1;0.8)
TNF-α	-0.5	-0.3	0.2	-0.6	-0.6	0.01	0.002	-0.1	-0.2	-0.2	0.1	-0.1
	(-1.9;1.0)	(-1.5;0.9)	(-2.1;2.4)	(-2.1;0.8)	(-2.6;1.3)	(-1.9;1.9)	(-0.4;0.4)	(-0.7;0.4)	(-0.7;0.3)	(-0.9;0.4)	(-0.5;0.8)	(-0.9;0.8)
Endothelial dysfunction												
sICAM-1	0.3	1.2	0.3	-0.7	0.2	**2.4**	0.1	-0.1	0.6	-0.4	-0.02	-0.2
	(-1.1;1.6)	(-0.1;2.5)	(-2.2;2.7)	(-2.5;1.1)	(-1.4;1.8)	**(0.6;4.2)**	(-0.3;0.6)	(-0.8;0.6)	(-0.2;1.4)	(-1.4;0.6)	(-0.5;0.5)	(-1.1;0.8)
vCAM-1	0.2	**1.2**	-0.4	1.2	1.1	1.2	0.2	-0.2	0.2	-0.2	0.3	-0.2
	(-1.2;1.6)	**(0.03;2.3)**	(-2.6;1.7)	(-0.4;2.8)	(-0.8;2.9)	(-0.4;2.8)	(-0.1;0.6)	(-0.7;0.3)	(-0.3;0.6)	(-0.8;0.5)	(-0.2;0.8)	(-0.9;0.7)
E-selectin	-1.0	-0.5	-0.3	-0.1	-1.4	-0.7	0.4	-0.2	0.7	-0.4	0.2	-0.3

	(-2.5;0.6)	(-1.7;0.7)	(-2.8;2.2)	(-2.0;1.8)	(-3.4;0.5)	(-2.3;0.9)	(-0.1;0.8)	(-0.7;0.4)	(-0.2;1.5)	(-1.1;0.4)	(-0.4;0.8)	(-1.1;0.6)
sTM	0.1	-0.3	1.0	-0.5	-0.2	-0.3	N/A	N/A	N/A	N/A	N/A	N/A
	(-1.0;1.3)	(-1.4;0.8)	(-0.9;2.8)	(-2.1;1.1)	(-1.7;1.2)	(-1.9;1.3)						

Results are expressed as unstandardized beta's with 95% confidence intervals. The model is adjusted for sex (for the total populations), time-varying covariates age, BMI, eGFR, hypertension, smoking status, medication use and CVD, and glucose metabolism status at baseline. In the Hoorn Study, the model is additionally adjusted for time-varying HbA1c. Significant effect modification by sex (P <0.10) is denoted with *.

Abbreviations: LVEF = left ventricular ejection fraction, LVMI = left ventricular mass index, LAVI = left atrial volume index, BMI = body mass index, eGFR = estimated glomerular function, CVD = cardiovascular diseases, CRP = C-reactive protein, SAA = serum amyloid A, IL-6 = interleukin-6, IL-8 = interleukin-8, sICAM1 = soluble intercellular adhesion molecule 1, TNFa = tumor necrosis factor α, sVCAM1 = soluble vascular adhesion molecule 1, sE-selectin = soluble endothelial selectin, sTM = soluble thrombomodulin.
