# Supplementary material for: Sex differences in the longitudinal relationship of low-grade inflammation and echocardiographic measures in the Hoorn and FLEMENGHO Study
Source: PLoS One. 2021 May 4;16(5):e0251148. doi: 10.1371/journal.pone.0251148 (PMC8096104; doi:10.1371/journal.pone.0251148)
Supplement: S3 Table — (PDF) [file pone.0251148.s003.pdf]

S3 Table. Sensitivity analyses of the ‘longitudinal’ and between person associations of low-grade inflammation or endothelial dysfunction on cardiac structure and function measures in the Hoorn Study and FLEMENGHO.

	Hoorn						FLEMENGHO					
	Total (N=289)		Female (N=137)		Male (N=152)		Total (N=315)		Female (N=159)		Male (N=156)	
LVEF, %	Within person	Between persons	Within person	Between persons	Within person	Between persons	Within person	Between persons	Within person	Between persons	Within person	Between persons
Low-grade inflammation without SAA												
Model 2	-0.3 (-2.5;1.9)	-0.5 (-2.0;1.1)	-1.6 (-4.8;1.7)	-0.6 (-2.7;1.5)	1.9 (-1.0;4.8)	-0.3 (-2.5;1.9)	0.02 (-1.3;1.3)	0.04 (-1.4;1.4)	0.5 (-1.5;2.4)	-0.3 (-2.1;1.5)	-0.6 (-2.4;1.2)	0.4 (-1.8;2.5)
Low-grade inflammation												
Model 2a	-0.5 (-2.6;1.6)	-0.5 (-2.1;1.1)	-1.0 (-4.4;2.3)	-0.5 (-2.7;1.8)	0.7 (-2.0;3.4)	-0.3 (-2.6;2.1)	0.1 (-1.2;1.3)	0.1 (-1.3;1.5)	0.5 (-1.4;2.4)	-0.3 (-2.2;1.5)	-0.6 (-2.3;1.2)	0.3 (-1.8;2.4)
Endothelial dysfunction without sTM												
Model 2	-0.4	0.6	-2.8	0.9	1.2	0.4	-0.6	-0.4	0.2	-0.7	**-1.2**	-0.2

	(-2.5;1.7)	(-0.6;1.9)	(-6.2;0.7)	(-1.1;3.0)	(-1.3;3.8)	(-1.3;2.0)	(-1.6;0.3)*	(-1.4;0.5)*	(-1.5;1.9)	(-2.1;0.8)	**(-2.3;-0.01)**	(-1.5;1.1)
Endothelial dysfunction												
Model 2a	-0.7<br>(-3.1;1.6)*	1.1<br>(-0.3;2.5)*	-3.6<br>(-7.4;0.2)	1.5<br>(-0.6;3.7)	0.8<br>(-2.0;3.6)	1.0<br>(-0.9;2.9)	-0.6<br>(-1.6;0.4)*	-0.4<br>(-1.4;0.5)*	0.3<br>(-1.4;2.0)	-0.7<br>(-2.1;0.8)	**-1.2**<br>**(-2.4;-0.1)**	-0.3<br>(-1.6;1.1)
LVMI, g/m$^{2.7}$	Total (N=304)		Female (N=146)		Male (N=158)		Total (N=477)		Female (N=242)		Male (N=235)	
	Within person	Between persons	Within person	Between persons	Within person	Between persons	Within person	Between persons	Within person	Between persons	Within person	Between persons
Low-grade inflammation without SAA												
Model 2	0.5<br>(-1.8;2.8)	-0.4<br>(-2.2;1.3)	0.8<br>(-2.2;3.9)	0.8<br>(-1.4;3.1)	0.4<br>(-3.0;3.8)	-2.0<br>(-4.7;0.7)	0.5<br>(-0.5;1.4)	-0.6<br>(-2.0;0.9)	0.4<br>(-0.9;1.7)	-0.2<br>(-2.0;1.6)	0.5<br>(-0.9;1.9)	-0.5<br>(-2.7;1.8)
Low-grade inflammation												
Model 2a	0.4<br>(-1.9;2.6)	-0.6<br>(-2.4;1.3)	0.6<br>(-2.5;3.7)	0.4<br>(-2.1;2.9)	0.1<br>(-3.1;3.3)	-1.6<br>(-4.3;1.1)	0.5<br>(-0.5;1.5)	-0.6<br>(-2.0;0.9)	0.4<br>(-0.9;1.7)	-0.2<br>(-2.0;1.6)	0.5<br>(-0.9;2.0)	-0.6<br>(-2.8;1.7)

Endothelial dysfunction without sTM Model 2	-0.5 (-2.7;1.9)	-0.3 (-1.7;1.2)	-1.5 (-4.8;1.9)	1.8 (-0.4;3.9)	0.7 (-2.4;3.8)	-1.6 (-3.6;0.4)	0.1 (-0.6;0.7)	-0.8 (-1.8;0.1)	0.2 (-0.8;1.3)	-0.7 (-2.0;0.7)	0.1 (-0.8;0.9)	-1.1 (-2.4;0.2)
Endothelial dysfunction Model 2a	0.8 (-1.8;3.4)	-0.6 (-2.2;0.9)	-0.2 (-4.0;3.5)	2.0 (-0.4;4.5)	2.1 (-1.5;5.7)	**-2.3 (-4.4;-0.1)**	0.1 (-0.6;0.7)	-0.8 (-1.8;0.1)	0.2 (-0.9;1.2)	-1.5 (-2.0;0.7)	-0.02 (-0.9;0.8)	-1.1 (-2.4;0.2)
	Total (N=318)		Female (N=150)		Male (N=168)		Total (N=484)		Female (N=245)		Male (N=238)	
LAVI, mL/m^2	Within person	Between persons	Within person	Between persons	Within person	Between persons	Within person	Between persons	Within person	Between persons	Within person	Between persons
Low-grade inflammation without SAA Model 2	-0.2 (-2.1;1.7)	0.8 (-1.2;2.7)	1.3 (-1.5;4.1)	-1.1 (-3.6;1.4)	-1.7 (-4.3;1.0)	2.3 (-0.7;5.2)	0.01 (-0.7;0.7)	-0.5 (-1.5;0.6)	0.02 (-0.8;0.9)	-0.6 (-1.9;0.6)	-0.1 (-1.2;0.9)	-0.3 (-2.0;1.4)
Low-grade inflammation												

Model 2a	-0.6 (-2.4;1.2)	0.9 (-1.1;2.9)	1.3 (-1.4;4.0)	-0.7 (-3.4;2.0)	-2.4 (-4.8;0.06)	1.9 (-1.0;4.8)	-0.01 (-0.7;0.7)	-0.5 (-1.6;0.6)	0.02 (-0.8;0.9)	-0.6 (-1.9;0.7)	-0.2 (-1.2;0.9)	-0.5 (-2.1;1.2)
Endothelial dysfunction without sTM												
Model 2	-0.2 (-2.1;1.7)	1.1 (-0.5;2.6)	-0.4 (-3.6;2.9)	0.4 (-1.9;2.8)	0.04 (-2.3;2.4)	1.4 (-0.7;3.6)	0.3 (-0.2;0.8)	-0.2 (-0.9;0.4)	0.5 (-0.2;1.2)	-0.4 (-1.4;0.5)	0.2 (-0.4;0.8)	-0.3 (-1.4;0.7)
Endothelial dysfunction												
Model 2a	-0.1 (-2.2;2.0)	0.7 (-1.1;2.4)	0.5 (-2.9;3.9)	0.1 (-2.4;2.6)	-0.3 (-2.8;2.3)	0.9 (-1.5;3.3)	0.3 (-0.2;0.8)	-0.2 (-0.9;0.5)	0.4 (-0.3;1.1)	-0.4 (-1.3;0.6)	0.2 (-0.4;0.8)	-0.4 (-1.4;0.7)

Results are expressed as unstandardized beta's with 95% confidence intervals. The model is adjusted for sex (for the total populations), time-varying covariates age, BMI, eGFR, hypertension, smoking status, medication use and CVD, and glucose metabolism status at baseline. In the Hoorn Study, the model is additionally adjusted for time-varying HbA1c. Significant effect modification by sex ($P < 0.10$) is denoted with *. Model 2a is adjusted just as model 2, but instead of medication use, adjusted for time-varying NSAID use. All models for the total populations are additionally adjusted for sex.

Abbreviations: LVEF = left ventricular ejection fraction, SD = standard deviation, BMI = body mass index, eGFR = estimated glomerular function, CVD = cardiovascular diseases, LVMI = left ventricular mass index, LAVI = left atrial volume index, NSAID = non-steroidal anti-inflammatory drug, SAA = serum amyloid A, sTM = soluble thrombomodulin.
