# Supplementary material for: Sex differences in the longitudinal relationship of low-grade inflammation and echocardiographic measures in the Hoorn and FLEMENGHO Study
Source: PLoS One. 2021 May 4;16(5):e0251148. doi: 10.1371/journal.pone.0251148 (PMC8096104; doi:10.1371/journal.pone.0251148)
Supplement: Attachment — Submitted filename: Response to Reviewers.docx [file pone.0251148.s004.docx]

**Ms. Ref. No.:** PONE-D-21-05358

**Title:** Sex differences in the longitudinal relationship of low-grade inflammation and echocardiographic measures in the Hoorn and FLEMENGHO Study

**Response to the editor and reviewers**

*We would like to thank the reviewers and editor very much for carefully reviewing our manuscript. Their review helped us to improve the manuscript. We have addressed all comments below, point-by-point. In addition, we have revised the manuscript and have highlighted the changes in yellow in the revised manuscript.*

**Editor:**

**E-mail 19th of April:**

*Reply: For the Hoorn Study, data are available from the Ethics Board of the Hoorn Studies (contact via* [*hoornstudy@vumc.nl*](mailto:hoornstudy@vumc.nl)*) for researchers who meet the criteria for access to confidential data. For FLEMENGHO, anonymized data can be made available to investigators for research based on a motivated request to be addressed to the Ethics board of the Hypertensive and Cardiovascular Epidemiology department via* [*infohypergenes@kuleuven.be*](mailto:infohypergenes@kuleuven.be)*. Data of both cohorts are stored on independent secured network locations within both hospitals. These network locations have daily back-ups.*

**E-mail 13th of April:**

1. Please provide non-author contact information for a data access committee, ethics committee, or other institutional body to which data requests may be sent.

*Reply: For the Hoorn Study, data are available from the Ethics Committee of the VU University Medical Center (contact via metc@vumc.nl) for researchers who meet the criteria for access to confidential data. For FLEMENGHO, anonymized data can be made available to investigators for research based on a motivated request to be addressed to the corresponding author via* [*jan.staessen@med.kuleuven.be*](mailto:jan.staessen@med.kuleuven.be)*. In Belgium the contact is via one of the co-authors as this person is also the contact person of the data access committee of FLEMENGHO.*

**E-mail 7th of April:**

1. Can the data set be fully anonymized and de-identified? If so, please upload the minimal anonymized data set necessary to replicate your study findings to a stable, public repository and provide us with the relevant URLs, DOIs, or accession numbers. Please see <https://eur04.safelinks.protection.outlook.com/?url=http%3A%2F%2Fwww.bmj.com%2Fcontent%2F340%2Fbmj.c181.long&amp;data=04%7C01%7Cs.remmelzwaal%40amsterdamumc.nl%7C00b2a0cf4fde42d90d7c08d8f9855441%7C68dfab1a11bb4cc6beb528d756984fb6%7C0%7C1%7C637533698333424813%7CUnknown%7CTWFpbGZsb3d8eyJWIjoiMC4wLjAwMDAiLCJQIjoiV2luMzIiLCJBTiI6Ik1haWwiLCJXVCI6Mn0%3D%7C2000&amp;sdata=%2BskNZlGCr9k7sw%2Fi%2BOhP%2BJgtDI1O7YfYaMF8mUTf0ic%3D&amp;reserved=0> for guidelines on how to de-identify and prepare clinical data for publication. For a list of recommended repositories, please see <https://eur04.safelinks.protection.outlook.com/?url=https%3A%2F%2Fjournals.plos.org%2Fplosone%2Fs%2Frecommended-repositories&amp;data=04%7C01%7Cs.remmelzwaal%40amsterdamumc.nl%7C00b2a0cf4fde42d90d7c08d8f9855441%7C68dfab1a11bb4cc6beb528d756984fb6%7C0%7C1%7C637533698333424813%7CUnknown%7CTWFpbGZsb3d8eyJWIjoiMC4wLjAwMDAiLCJQIjoiV2luMzIiLCJBTiI6Ik1haWwiLCJXVCI6Mn0%3D%7C2000&amp;sdata=fatYeuMqftCCa2ZD8w6%2B4E5%2FWhwzFi3lx91%2Bmp0SBRs%3D&amp;reserved=0>. You also have the option of uploading the data as Supporting Information files, but we would recommend depositing data directly to a data repository if possible.

We will update your Data Availability statement on your behalf to reflect the information you provide.

*Reply: Unfortunately, we cannot share our data publicly, even after de-identification of all data. In these ‘older’ cohorts we did not ask specifically for consent to share the participants’ data. We only have consent to share their de-identified data with researchers after approval of the boards of the Hoorn studies and FLEMENGHO, respectively. As stated in the additional information, researchers can hand in their research plans at the respective boards.*

1. Please ensure that your manuscript meets PLOS ONE's style requirements, including those for file naming. The PLOS ONE style templates can be found at <https://journals.plos.org/plosone/s/file?id=wjVg/PLOSOne_formatting_sample_main_body.pdf> and <https://journals.plos.org/plosone/s/file?id=ba62/PLOSOne_formatting_sample_title_authors_affiliations.pdf>

*Reply: We renamed all supplemental files accordingly and updated the reference list according to the journal’s standards.*

2. In ethics statement in the manuscript and in the online submission form, please provide additional information about the patient records/samples used in your retrospective study. Specifically, please ensure that you have discussed whether all data/samples were fully anonymized before you accessed them.

*Reply: We added the following information in the Methods (page 7, lines 160-161): ‘All data and samples from the study participants were fully anonymized before analyses.’*

**Reviewer #1:**

It is difficult to determine the exact echocardiographic methods employed. The references then reference another paper that references another paper, and I am not sure if the original reference is to support the use of the echocardiographic measurement or the methodology used. A few sentences would make this clearer to the reader. Suggestions are as follows using sentences copied from the echocardiographic methodology on page 8:

LVEF (%) was used as an index of left ventricular systolic function and was calculated by the modified Simpson’s rule(26) (Please specify single plane apical 4 chamber view or biplane apical 4 and 2 chamber views).

Left ventricular mass (Please state if this was done using M-Mode or 2D imaging, I think it was M-Mode, but I can't be sure) –indexed to height to the power of 2.7 (LVMI, g/m^2.7^) – was determined to assess cardiac structure.

LAVI (mL/m2) served as an index for LV diastolic function and was calculated by indexing single-plane maximum left atrial volume (please state apical 4 chamber view, if that was the view used) by body surface area.

The same methodology section states that mitral valve disease was excluded, which I found in the paper referenced, but I can't tell if aortic valve disease was also excluded. Please clarify.

*Reply: We added the following details in the Methods (page 8, lines 191-199, and page 9, lines 200-201): ‘LVEF (%) was used as an index of left ventricular systolic function and was calculated from the apical four chamber view in the Hoorn study[24, 25] and apical four- and two-chambers views in FLEMENGHO[27], using the modified Simpson’s rule[28]. Left ventricular mass – measured in M-Mode and indexed to height to the power of 2.7 (LVMI, g/m^2.7^) – was determined to assess cardiac structure. LAVI (mL/m2) served as an index for LV diastolic function and in the Hoorn study was calculated by indexing single-plane maximum left atrial volume from the apical four chamber view by body surface area. In FLEMENGHO, left atrial dimensions were measured in 3 orthogonal planes (parasternal long, lateral, and supero-inferior axes) and LAVI was calculated using the prolate-elipsoid method[29] and was indexed to body surface area.’*

*In the current study we did not exclude on additional diseases, such as mitral valve disease, as those patients with valvular diseases were already excluded because of missing data in either inflammatory or echocardiographic measurements.*

***References***

*24. van den Hurk K, Alssema M, Kamp O, Henry RM, Stehouwer CD, Diamant M, et al. Slightly elevated B-type natriuretic peptide levels in a non-heart failure range indicate a worse left ventricular diastolic function in individuals with, as compared with individuals without, type 2 diabetes: the Hoorn Study. European Journal of Heart Failure. 2010;12(9):958-65. doi: 10.1093/eurjhf/hfq119.*

*25. Henry RM, Paulus WJ, Kamp O, Kostense PJ, Spijkerman AM, Dekker JM, et al. Deteriorating glucose tolerance status is associated with left ventricular dysfunction--the Hoorn Study. The Netherlands journal of medicine. 2008;66(3):110-7. Epub 2008/03/20. PubMed PMID: 18349466.*

*27. Kloch-Badelek M, Kuznetsova T, Sakiewicz W, Tikhonoff V, Ryabikov A, Gonzalez A, et al. Prevalence of left ventricular diastolic dysfunction in European populations based on cross-validated diagnostic thresholds. Cardiovasc Ultrasound. 2012;10:10. doi: 10.1186/1476-7120-10-10. PubMed PMID: 22429658; PubMed Central PMCID: PMCPMC3351014.*

*28. Folland ED, Parisi AF, Moynihan PF, Jones DR, Feldman CL, Tow DE. Assessment of left ventricular ejection fraction and volumes by real-time, two-dimensional echocardiography. A comparison of cineangiographic and radionuclide techniques. Circulation. 1979;60(4):760-6. doi: 10.1161/01.CIR.60.4.760.*

*29. Lang RM, Badano LP, Mor-Avi V, Afilalo J, Armstrong A, Ernande L, et al. Recommendations for Cardiac Chamber Quantification by Echocardiography in Adults: An Update from the American Society of Echocardiography and the European Association of Cardiovascular Imaging. European Heart Journal - Cardiovascular Imaging. 2015;16(3):233-71. doi: 10.1093/ehjci/jev014.*

**Reviewer #2:**

I read article entitled “Sex differences in the longitudinal relationship of 2 low-grade inflammation and echocardiographic 3 measures in the Hoorn and FLEMENGHO Study”. I want to thank you for this good work. They did not show consistent associations of markers of low-grade inflammation or endothelial dysfunction with cardiac structure and function, nor any evidence of effect modification by sex. The authors could make diastolic function assessment in accordance with ASE / EACVI standards. However, it is a well written study. This paper is suitable for publication in its current form.

*Reply: We thank the reviewer for their feedback. As we did not measure e’ at baseline and TR velocity at both baseline and follow-up, assessment of diastolic function in accordance with ASE / EACVI standard could not be possible in our two population-based cohorts.*
